# Phenotypic Plasticity of Cancer Cells Based on Remodeling of the Actin Cytoskeleton and Adhesive Structures

**DOI:** 10.3390/ijms22041821

**Published:** 2021-02-12

**Authors:** Svetlana N. Rubtsova, Irina Y. Zhitnyak, Natalya A. Gloushankova

**Affiliations:** Institute of Carcinogenesis, N.N. Blokhin National Medical Research Center of Oncology, 24 Kashirskoye Shosse, 115478 Moscow, Russia; gaart2@gmail.com (S.N.R.); irazhitnyak@gmail.com (I.Y.Z.)

**Keywords:** cancer cells, EMT, plasticity, migration, actin cytoskeleton, E-cadherin, adherens junctions

## Abstract

There is ample evidence that, instead of a binary switch, epithelial-mesenchymal transition (EMT) in cancer results in a flexible array of phenotypes, each one uniquely suited to a stage in the invasion-metastasis cascade. The phenotypic plasticity of epithelium-derived cancer cells gives them an edge in surviving and thriving in alien environments. This review describes in detail the actin cytoskeleton and E-cadherin-based adherens junction rearrangements that cancer cells need to implement in order to achieve the advantageous epithelial/mesenchymal phenotype and plasticity of migratory phenotypes that can arise from partial EMT.

## 1. Introduction

Despite improvements in protocols of radio-, chemo-, and immunotherapy, distant metastases are still responsible for the great majority of cancer-related deaths. Detailed studies of mechanisms of cancer cell dissemination are of great importance for understanding tumor progression and developing new targeted drugs. In this Review, we present current knowledge on how cancer cells acquire the ability to escape from primary tumor, adapt their behavior to changes in their microenvironment during metastatic dissemination and forming secondary (metastatic) tumors in distant organs or lymph nodes. Along with persistent cell proliferation and apoptosis suppression, one of the major characteristics of tumor cells is their plasticity which allows them to switch between different modes of migration and support their survival, which results in successful metastatic colonization. Recent data suggest that cancer cells expressing both epithelial and mesenchymal markers maintain a high degree of plasticity, can survive in ectopic environments, exhibit a heightened resistance to chemotherapy and have a high tumor initiating and metastatic potential. Cells with a hybrid epithelial/mesenchymal phenotype are likely to be playing a major role in cancer progression.

## 2. Epithelial Cells

**Figure 1:**
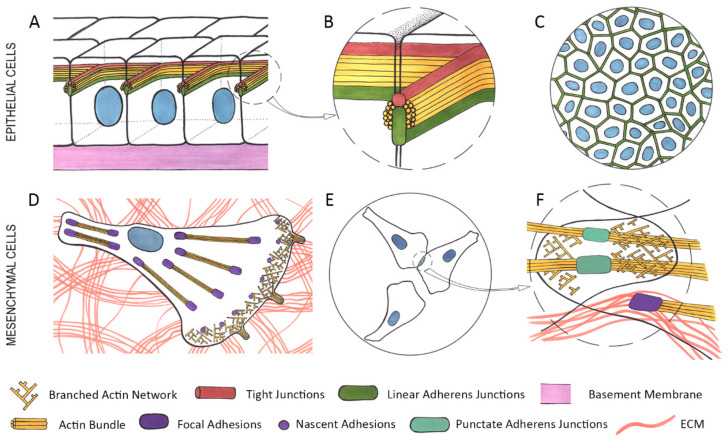
Organization of the actin cytoskeleton and adhesive structures in epithelial and mesenchymal cells. (**A**)—a monolayer of epithelial cells. (**B**)—a close-up of an area in the dashed circle on A—stable cell-cell adhesion in epithelial cells is provided by apical adhesion belts comprised by tight junctions (TJs) (red) and linear adherens junctions (AJs) (green), both of which are closely associated with the circumferential actin bundle (yellow). (**C**)—a top view of a monolayer of epithelial cells, connected by stable linear AJs. (**D**)—a mesenchymal cell exhibiting branched actin network (yellow) and nascent focal adhesions (FAs) (purple) in lamellipodia at the leading edge. Closer to the center of the cell and in the rear are mature FAs (purple) associated with straight actin bindles (yellow). Both nascent and mature FAs are connected to the extracellular matrix (ECM) (pink). (**E**)—an area of cell-cell interaction between motile mesenchymal cells. (**F**)—a close-up of the area in the dashed circle on E—overlapping lamellae containing branched actin network (yellow) point to the lack of contact paralysis, unstable punctate AJs (green) are associated with straight actin bundles. Mature FAs (purple) connected to the ECM (pink) are associated with straight actin bundles.

Most tumors in adults are carcinomas which arise from epithelial tissues. Epithelial tissues are organized into layers composed of non-motile cells tightly connected by adhesive structures (adherens junctions (AJs), tight junctions (TJs) and desmosomes) with adjacent cells, and stably attached to the underlying basement membrane (BM) via hemidesmosomes (Figure 1A–C) [1]. Epithelial cells exhibit apical–basal polarity of membrane domains, protein complexes, and cytoskeletal components. TJs and AJs form the apical junctional complex [1]—a continuous belt around the apical part of cell—which is associated with the circumferential actin bundle (Figure 1A,B). TJs define the boundary between the apical and basolateral domains in epithelial cells. TJs form a lateral diffusion barrier between the apical and basolateral domains. TJs are composed of occludin, claudins, and JAMs (Junctional Adhesion Molecules) that are linked to the actin cytoskeleton through ZO (zonulae occludens) proteins [2]. Apical-basal polarity is controlled by: (1) the apical complex—the PAR proteins PAR3 and PAR6, aPKC, the CDC42 GTPase, the CRUMBS complex (CRUMBS, PALS1, PATJ, and LIN-7); (2) the basolateral complex (SCRIB, DLG, LGL); and (3) a cytoplasmic group of polarity proteins—PAR4/LKB1, PAR1/MARK, PAR5/14-3-3 [3]. Multi-level regulatory interactions between polarity proteins are essential for establishing and maintaining cell polarity.

**Figure 2:**
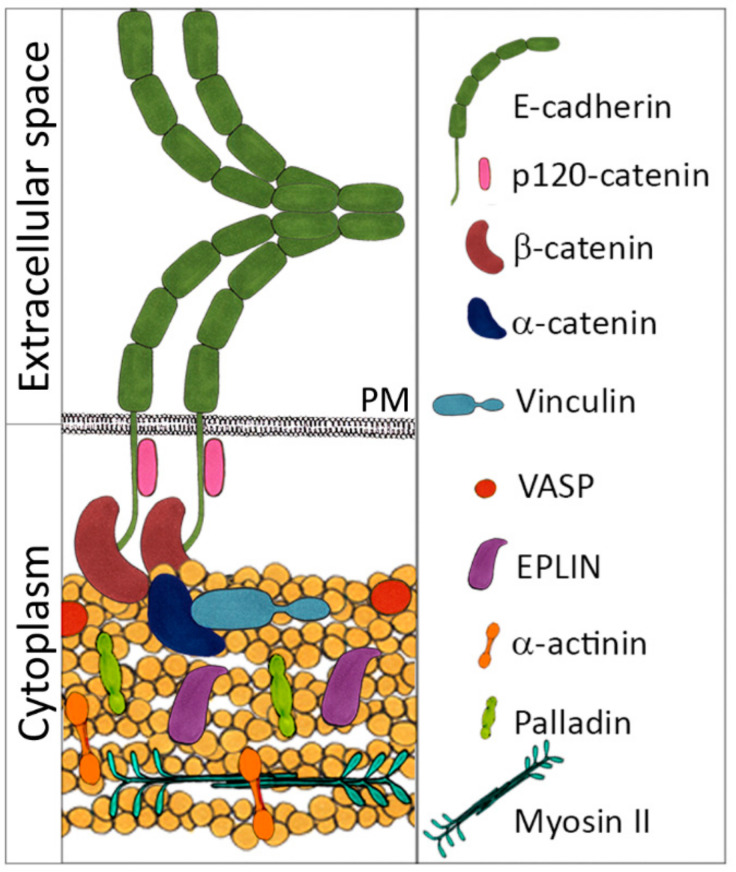
Structure and composition of an epithelial adherens junction. E-cadherin molecules (green) on the surface of adjacent cells connect with one another via their extracellular domains. Below the cytoplasmic membrane (PM), the intracellular domain of E-cadherin interacts with p120 (pink) and β-catenins (dull red). β-catenin binds to α-catenin (dark blue), which, in turn, interacts with vinculin (blue) and directly with actin filaments (yellow). Vinculin binds to actin filaments to stabilize the cadherin-catenin complex. Various actin-binding proteins such as VASP (bright red), EPLIN (Epithelial Protein Lost in Neoplasm) (purple), α-actinin (orange), palladin (light green) and myosin II (cyan-green) are associated with junctional actin.

AJs are particularly important for epithelial tissue integrity as they provide strong calcium-dependent cell-cell adhesion. In non-tumorigenic epithelial cells and in carcinoma cells that maintain the epithelial phenotype, AJs are organized linearly into zonulae adherens (adhesion belt), located in the apical junctional complex just below TJs (Figure 1) [1]. These linear AJs are very stable and dissolve only during mitosis. Disruption of AJs results in loss of cell–cell adhesion and dissociation of the cells. In epithelial cells, AJs are formed by transmembrane E-cadherin adhesion receptors whose cytoplasmic domains bind to members of the catenin protein family, β-catenin and p120 (Figure 2) [4,5]. β-catenin interacts with the N-terminal domain of α-catenin, the central part of the α-catenin molecule contains the vinculin-binding domain, and α-catenin’s C-terminal domain directly binds actin filaments [6,7]. Contractile forces generated by actin-coupled myosin II induce unfolding of the actin-binding domain of α-catenin, which enhances actin binding [8]. Force-dependent destabilization of the interactions between MI vinculin-binding and MII and MIII inhibitory domains of α-catenin leads to opening of the MI domain [7,9] that results in a significant increase in its affinity for vinculin, which, in turn, recruits additional actin that stabilizes the cadherin/catenin complex. This junctional actin, tightly associated with the circumferential actin bundle, is crucially important for the assembly and maintenance of AJs [10,11].

Super resolution microscopy of Madine-Darby canine kidney (MDCK) epithelial cells cultured on an E-cadherin-coated planar substrate demonstrated that E-cadherin-based adhesions of the cells maintained a strict compartmentalized architecture. The cadherin-catenin compartment containing E-cadherin and catenins and the actin cytoskeletal compartment containing actin cytoskeleton and actin-binding proteins (EPLIN (Epithelial Protein Lost in Neoplasm), myosin II, palladin, and α-actinin) sandwiched the intermediate zone containing vinculin and VASP [12]. EPLIN additionally stabilizes the circumferential actin bundle by inhibiting actin depolymerization and crosslinking actin filaments [13]. SiRNA-mediated knockdown of EPLIN results in disappearance of circumferential actin bundles and converting linear AJs into punctate AJs associated with radial actin bundles [14]. Both myosin IIA and IIB isoforms are important for establishment and maintenance of linear AJs. Actomyosin contractility defines the morphology of linear AJs, drives compaction of epithelial cells and supports integrity of epithelial layers. Myosin IIA is required for assembly and maintenance of junction complexes [15]. Myosin IIA depletion disrupts formation of E-cadherin junctional belt in the apical part of epithelial cells. Myosin IIB depletion decreases actin content in circumferential actin bundles associated with linear AJs [16,17]. As was observed in 2D cultures of epithelial cells, AJ maturation required activity of the Rho family of GTPases which recruited formins promoting elongation of linear actin filaments [10]. mDIA1, recruited by active RAC1 [18], and FMNL2, recruited by active RHOA [19], are necessary for E-cadherin stabilization in AJs. 

Analyzing collisions between epithelial cells, we described the phenomenon of contact paralysis of protrusive activity that was fundamentally different from the behavior of mesenchymal cells in response to cell-cell interactions [20,21,22]. A collision between epithelial cells resulted in dramatic cessation of protrusive activity in the zone of the expanding cell-cell contact (contact paralysis) whereas after the initial cell-cell contact mesenchymal cells continued formation of protrusions in the zone of cell-cell collision (Figure 1E,F) [20,23]. Contact paralysis in epithelial cells is caused by tangential tension at the cell-cell boundaries generated by actin-myosin bundles [24]. Live cell imaging and analysis of the spatiotemporal regulation of RAC1 and RHOA activity and actomyosin contractility during de novo formation of cell–cell adhesions detected high RAC1 activity only at the early stages of formation of stable AJs during initial lamellipodia interactions, but it was down-regulated during lateral expansion of linear AJs, while RHOA and actomyosin contractility were activated at the edges of expanding contact [25]. Contact paralysis is essential for maintenance of stable cell-cell adhesion. Its loss during neoplastic transformation may facilitate cancer cell dissemination during metastasis. 

## 3. Epithelial-Mesenchymal Transition (EMT)

Epithelial cells can acquire mesenchymal phenotypes using a program known as the epithelial–mesenchymal transition (EMT). While undergoing EMT, epithelial cells lose apical-basal polarity and stable cell-cell adhesion and acquire migratory activity. EMT is implicated in various biological processes such as embryonic development, tissue repair, wound healing and pathological conditions such as tissue/organ fibrosis and cancer progression. The bulk of carcinoma cells in primary tumors exhibit epithelial characteristics but in order to metastasize, carcinoma cells undergo EMT to acquire a more mesenchymal phenotype that will allow them to detach from neighboring cells, overcome tissue barriers and migrate through tissues. During EMT, carcinoma cells lose their apical-basal polarity; stable E-cadherin-based AJs and the curcumferential actin bundle supporting their integrity become disrupted; de novo actin polymerization leads to appearance of dynamic lamellipodia which are the engine of cancer cell migratory and invasive activity. Cells acquire front-rear polarity and become capable of directional migration. 

EMT is the most widely studied example of phenotypic plasticity, and its contribution to promoting cancer cell invasion and metastasis has been established for many types of carcinomas [26,27,28,29]. The most common sequence of events nesessary to successfully colonize distant organs is termed “the invasion-metastasis cascade” and includes the following steps: (1) invasion at the local site through BM; (2) migration in surrounding tissues; (3) entrance into a circulatory system (blood or lymph vessel) and traveling through circulation; (4) arrest at a distant site and exit from the circulatory system; (5) survival and proliferation in a distant organ, resulting in formation of micro- and macrometastases [30]. In cancer, EMT is activated by signaling pathways from TGFβ (Transforming Growth Factor Beta), EGF (Epidermal Growth Factor), HGF (Hepatocyte Growth Factor), Notch, FGF (Fibroblast Growth Factor), Wnt, and IGF (Insulin-Like Growth Factor), signals from tumor microenvironment (e.g., cancer-associated macrophages or fibroblasts), hypoxia and increased matrix stiffness [29,31,32,33,34].

In many cancer types these signals activate core EMT-inducing transcription factors (EMT-TFs)—SNAIL, SLUG, TWIST1, ZEB1, and ZEB2 via transcriptional and post-transcriptional mechanisms [35]. These EMT-TFs are considered the key drivers of cancer progression, and their high expression has been detected in many invasive carcinomas [36,37,38,39,40]. EMT-TFs down-regulate the expression of genes associated with the epithelial phenotype (e.g., E-cadherin, occludin, cytokeratins, polarity genes) and induce the expression of genes that sustain the mesenchymal phenotype (*N*-cadherin, vimentin, fibronectin, and certain integrins) and matrix metalloproteinases (MMPs) (Table 1) [31,41]. Expression of mesenchymal markers in cells of epithelial origin may be advantageous for cell migration. *N*-cadherin increased migration and invasion of breast cancer cells regardless of E-cadherin expression [42,43]. Vimentin, through its interaction with RhoGEFs, could promote cell migration by influencing structure and dynamics of the actin cytoskeleton [44]. Vimentin binding to the cytoplasmic tail of β3 integrin could directly affect integrin-mediated signaling in the cell [45]. However, the mechanisms regulating vimentin involvement in controlling cell migration require further studies.

EMT-TFs play essential roles in cancer cell migration, invasion and metastasis. Studies in vitro demonstrated that exogenous SNAIL and SLUG increased migratory and invasive capacity of cancer cells [47,60,75]. SNAIL promoted collective migration in squamous carcinoma cells by inducing the expression of claudin-11 [57]. ZEB1 and ZEB2 induced EMT in epithelial cells and promoted cell migration and invasion in breast and colorectal cancer cells [38,73,76]. ZEB1 promoted metastasis in the KPC mouse model of pancreatic cancer [77]. TWIST1 expression enhanced cell motility in hepatocellular carcinoma cells [64]. Expression of TWIST1 correlated with lymph node metastasis of breast cancer [37]. 

Besides promoting metastatic dissemination, the EMT program and EMT-TFs appear to serve as major drivers of cancer progression. EMT-TFs such as SNAIL and SLUG can activate and maintain stemness traits in carcinoma cells as was shown for mammary and thyroid carcinoma respectively [78,79]. EMT-TFs have been shown to promote DNA damage repair and radioresistance [80,81]. Emerging evidence suggests that EMT contributes to increased cell survival, suppression of apoptosis and resistance to chemotherapy and immunotherapy [31,35,82,83].

## 4. Hybrid Epithelial-Mesenchymal Phenotype 

During the last decade it has been revealed that in cancer, EMT is not a binary switch between epithelial and mesenchymal states, but a process which, depending on particular combinations of intrinsic and extrinsic factors, generates subpopulations of cells in various intermediate states between the epithelial and mesenchymal phenotypes [26,27,84]. Carcinoma cells frequently undergo partial EMT (pEMT) by acquiring mesenchymal traits while retaining epithelial markers. Cells possessing the hybrid epithelial/mesenchymal phenotype retain expression of cytokeratins or E-cadherin or EpCAM (Epithelial Cell Adhesion Molecule) while inducing a mesenchymal marker vimentin and, in a substantial number of cases, N-cadherin [26,29,78,83,85,86,87,88]. Cells undergoing pEMT switch from stable linear AJs to unstable punctate AJs. The robust circumferential actin bundle is replaced with dynamic lamellipodia in the front and straight actin bundles assosiated with AJs and focal adhesions. Cells with the hybrid epithelial/mesenchymal phenotype acquire migratory activity [22]. 

EMT in cancer exhibits great diversity and is a local and dynamic process [78,89]. It is considered that the hybrid epithelial/mesenchymal phenotype is a plastic state, prone to changes depending on the cell’s microenvironment. This phenotype, which allows the cell to quickly adapt and change its morphology and migratory properties accordingly is especially advantageous during metastatic colonization [84,86,90,91]. Single-cell transcriptomic analysis of genes associated with stemness (OCT4 and SOX2) and EMT (SNAI2, SKP2 and TWIST1) in mouse models of human triple negative breast cancer demonstrated higher expression of these genes in early stage metastatic disease than either in primary tumors or in advanced stage metastatic disease [92]. Using multicolour fluorescence-activated cell sorting and single cell RNA sequencing, various subpopulations of hybrid phenotype cells expressing different combinations of epithelial and mesenchymal markers were identified in mouse models of skin and breast carcinoma [84]. Intravital microscopy of a breast cancer model harboring an EMT-driven color switch revealed a population of tumor cells undergoing EMT at the boundaries of tumor lobules adjacent to the blood vessel-enriched stroma [93]. In a lineage-labelled mouse model of pancreatic ductal adenocarcinoma, it was observed that tumor cells undergoing the pEMT program migrated as clusters and exhibited epithelial-mesenchymal plasticity [94]. Single-cell transcriptomic analysis of cells from head and neck squamous cell carcinoma patients showed that cells exhibiting the pEMT program spatially localized at the leading edges of primary tumors in contact with cancer-associated fibroblasts (CAFs) [95]. EMT may be regulated by growth factors secreted by the cells from the tumor microenvronment. It was shown that CAFs secreting HGF and TGFβ promoted activation of carcinoma cells migration [96,97]. In another study, migration and intravasation of tumor cells was induced by tumor-associated macrophages producing EGF [98,99].

A significant amount of data accumulated in recent years shows that cells with the hybrid epithelial/mesenchymal phenotype exhibit high metastatic potential [27,83,84,100,101]. Intravenous injection of different subpopulations of squamous cell carcinoma cells derived from hair follicles or prostate carcinoma cells demonstrated increased lung metastasis of pEMT tumor cells as compared to cells with mesenchymal phenotype [85,102]. Analysis of circulating tumor cells (CTCs) originated from the primary tumor and travelling through the bloodstream has provided important insights into phenotypic traits of disseminating cancer cells. CTCs displaying both epithelial (e.g., E-cadherin, cytokeratins) and mesenchymal markers (e.g., vimentin) have been found in the blood of patients with breast, lung, colon, prostate, and liver cancers [90,103,104,105]. RNA profiling of breast cancer CTCs has revealed the existence of different EMT states [90]. The presence of expression of epithelial markers in CTCs is associated with better survival of cancer cells and poorer clinical prognosis. In prostate cancer patients, detection of CTCs expressing high levels of EpCAM, correlated with poor survival. CTCs with low levels of EpCAM did not affect survival of the patients [106]. In another study, the presence of CTCs with the epithelial/mesenchymal phenotype, co-expressing cytokeratin, high levels of ALDH1, and nuclear TWIST1, in the blood of metastatic breast cancer patients had a significant negative prognostic value [107]. 

In the blood of patients with breast, lung and head and neck cancer along with single CTCs, CTC clusters were also found [90,108,109,110]. CTCs obtained from patients with various types of cancers were often joined into clusters by E-cadherin-based AJs. These clusters were more resistant to anoikis in the bloodstream and more effective at metastatic outgrowth in distant organs [111,112]. To transit through capillary vessels, CTC clusters of ≤20 cells could reversibly stretch into single-file chains [113]. Association of clusters with platelets could protect cancer cells from elimination by immune system [27]. Studies of patients with lung, breast and head and neck cancer demonstrated that CTC clusters were associated with poorer prognosis as compared to single CTCs [108,109,110]. 

## 5. Role of E-Cadherin in Carcinoma Cell Dissemination

For a long time, it was an accepted fact that down-regulation of E-cadherin expression plays a key role in carcinoma progression by promoting invasion and metastasis [114,115]. Loss of E-cadherin expression has been found in esophageal, gastric, breast, colon, prostate, and liver cancer [116,117,118,119,120,121]. It is clearly apparent that down-regulation of E-cadherin expression leads to destabilization of AJs and facilitates the initial dissociation of cells from the primary tumor. Tumor-suppressive role of E-cadherin may also be explained by its negative role in regulation of the canonical WNT/β-catenin pathway through sequestering β-catenin at the cell membrane [122]. Additionally, E-cadherin localized at the membrane in AJs can negatively regulate the ligand-dependent activation of EGFR, IGF-1R, and c-Met [123,124]. E-cadherin in the apical zonulae adherens has been found to recruit DROSHA and DGCR8, the core components of the RNA-induced silencing complex (RISC) and various mRNAs and miRNAs including miRNA-24 and miRNA-200c, via PLEKHA7. MiRNA-24 and miRNA-200c associated with zonulae adherens down-regulate expression of the oncogenes MYC and JUN, and a pluripotency factor SOX2 by silencing their mRNAs [125].

**Figure 3:**
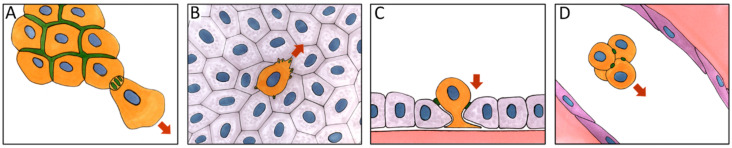
E-cadherin-based adherens junctions (AJs) facilitate cancer cell dissemination. (**A**)—collective invasion. (**B**)—migration over a monolayer of normal epithelial cells. (**C**)—invasion of the monolayer of normal epithelial cells. (**D**)—a circulating tumor cell (CTC) cluster traveling through circulation. Orange—cancer cells, grey—normal epithelial cells, purple—endothelial cells. Green—E-cadherin-based AJs, red arrows—direction of cell migration.

Although E-cadherin has been considered a tumor suppressor, accumulating evidence suggests a more complicated role of E-cadherin in cancer cell biology. A wealth of histochemical data points to retention of E-cadherin expression in many invasive carcinomas and their metastases (e.g., ductal breast, colorectal, prostate, pancreas carcinoma and oral squamous cell carcinoma) [126,127,128,129,130,131]. Well-differentiated cells that maintain epithelial morphology and apico-basal polarity form the bulk of many early stage carcinomas [31]. In vitro, cells of various carcinomas (e.g., MCF-7 and T-47D breast carcinoma, A-549 lung carcinoma, HT-29, Caco-2 and T84 colon carcinoma cell lines) are capable of maintaining stable linear AJs associated with the circumferential actin bundles. Many types of carcinomas (e.g., breast, colorectal, prostate, oral squamous cell carcinoma) can invade as multicellular groups in which cells remain attached to the neighbors by E-cadherin-based AJs (collective invasion) (Figure 3A) [132,133,134,135]. Collective invasion may be facilitated by CAFs connected with cancer cells by heterophilic E-N-cadherin AJs. CAFs may act as leader cells and remodel ECM creating migration tracks for follower cancer cells [136,137].

As was mentioned earlier, E-cadherin may contribute to dissemination of cancer cells through circulation by helping form CTC clusters (Figure 3D). Using mouse and human models of luminal and basal invasive ductal breast carcinomas, it has been recently demonstrated that E-cadherin contributes to metastasis by acting as a survival factor for cancer cells [111,112]. Loss of E-cadherin reduced proliferation and survival of CTCs and their dissemination via TGFβ-stimulated accumulation of reactive oxygen species and induction of apoptosis [138]. 

Although cancer cells initiating the invasion-metastasis cascade acquire a more mesenchymal phenotype, reversal to the epithelial phenotype (mesenchymal-epithelial transition, MET) and re-expression of E-cadherin are important for metastatic colonization. Earlier, immunohistochemical studies of invasive breast cancer have detected higher levels of E-cadherin expression in metastases than in the primary tumor [139]. Direct intravital microscopy in mice demonstrated that carcinoma cells spontaneously undergoing EMT and acquiring migratory activity reverted to epithelial phenotype in growing metastases [85,89]. It was shown that EMT-TFs TWIST1 and PRRX1 (Paired Related Homeobox 1) that were necessary for initiation of metastasis, needed to be repressed for successful metastatic outgrowth [140,141,142]. In a mouse breast cancer model it was recently shown that metastatic colonization by cancer cells required a metastatic niche activation in distant organs that promoted the shift toward a more epithelial phenotype and up-regulation of E cadherin expression [143,144]. Taken together, these data point at extreme importance of E-cadherin-based AJs in cancer cell dissemination.

We were first to show that E-cadherin-based AJs in neoplastically transformed epithelial cells undergoing pEMT were different from the linear AJs found in normal epithelial cells. Using a panel of chemically or oncogenically transformed IAR rat liver epithelial cells, we observed radial (punctate) AJs, which behaved radically differently from the stable linear AJs in normal epithelial cells [22]. These punctate AJs were associated with straight actin bundles and were very dynamic and unstable much like the N-cadherin-based AJs of bona fide mesenchymal cells. Formation and maintenance of punctate AJs depended on myosin II-mediated contractility, as both the ROCK inhibitor Y-27632 and the myosin II ATPase inhibitor blebbistatin reduced mature punctate AJs to nascent dot-like AJs. Transformed epithelial cells with these punctate AJs were capable of effective collective migration on 2D adhesive substrates and in migration chambers [145]. We also found that neoplastically transformed epithelial cells retaining E-cadherin expression could form E-cadherin-based AJs with normal epithelial cells. Expression of E-cadherin and its assembly into dynamic punctate AJs allowed transformed cells to migrate over epithelial monolayer and to invade the monolayer (Figure 3B,C) [146]. When the formation of punctate AJs in transformed cells was abolished by expression of either a dominant negative E-cadherin construct or an anti-E-cadherin siRNA, migration over and invasion of the epithelial monolayer considerably decreased. Depletion of N-cadherin did not have any effect on invasive behavior of transformed cells. Thus, cancer cell dissemination may be dependent on formation of E-cadherin-based cell-cell contacts between cancer cells and the surrounding normal cells.

In a recent work by Indra et al. spatial organization of punctate AJs in A-431 carcinoma cells was studied in great detail. It was shown that punctate AJs consisted of dense, paracrystalline nanoclusters formed through cis and trans interactions of cadherin ectodomains, interspersed with less dense cadherin regions [147]. It was also shown that F-actin at punctate AJs consisted of two different structurally distinct regions—stable bundle stalk enriched with calponin and highly dynamic AJ proximal region of branched F-actin at the tip of the bundle enriched with VASP and F-actin depolymerization factor, cofilin-1. The assembly and disassembly of both F-actin and cadherin clusters were tightly coupled [148].

## 6. Rearrangement of the Cytoskeleton and Adhesive Structures in Cells Undergoing EMT

**Figure 4:**
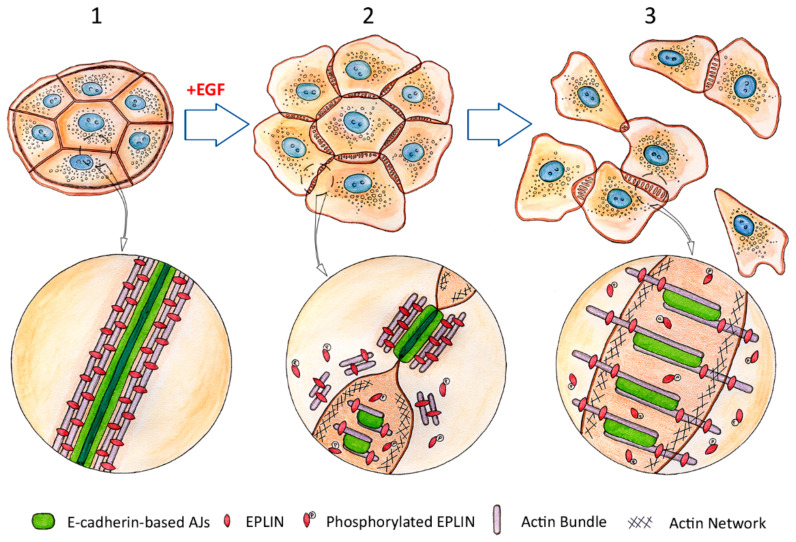
Dissolution of the circumferential actin bundle, degradation of EPLIN and rearrangement of E-cadherin-based adherens junctions (AJs) during the early stages of epithelial-mesenchymal transition (EMT). Adapted from Zhitnyak et al., Cells 2020, 9, 578 [150]. Top row: progression of EMT induced by Epidermal Growth Factor (EGF). **1**—epithelial cells before treatment with EGF. An island of non-motile cells tightly connected by linear AJs. Circled area in higher magnification below: a stable linear AJ between epithelial cells, associated with a robust circumferential actin bundle. EPLIN supports the bundle integrity by cross-linking actin filaments. **2**—early stages of EGF-induced EMT (5–10 min). Protrusive activity increases at the cell edges and protrusions begin to form at the cell-cell boundaries. Contact paralysis at the cell-cell boundaries disappears. Linear AJs are partially disassembled and replaced by punctate AJs. Circled area in higher magnification below: Dissolution of the circumferential actin bundle and reorganization of linear AJs. Part of the AJ still maintains its original linear configuration and is associated with the remnants of the circumferential actin bundle. Phosphorylation of EPLIN results in its detachment from the circumferential actin bundle which leads to disintegration of the bundle. Bottom part—formation of small punctate AJs associated with nascent straight actin bundles. **3**—later stages of EGF-induced EMT (15–60 min). Cells acquire migratory properties and detach from each other, breaking cell-cell adhesion. The new punctate AJs formed by migratory cells are dynamic and unstable. Circled area in higher magnification below: mature punctate AJs during later stages of EMT. The AJs are longer than in 2 and are associated with thicker straight actin bundles again fortified by EPLIN.

While undergoing EMT, cells have to reorganize their cytoskeleton to weaken cell-cell adhesion and acquire efficient directional motility. As mentioned earlier, weakening of cell-cell adhesion may be connected with transcriptional repression of the CDH1 gene that encodes E-cadherin or loss of surface E-cadherin through protein endocytosis [91,149]. The earliest stages of EMT leading to disruption of cell-cell contacts that allows a cell to escape from neighboring cells remained unexplored until now. Recently, using live cell imaging, we analyzed early events during EMT induced by EGF in IAR-20 rat liver epithelial cells (Figure 4) [150]. We detected rapid (within 5–10 min) fragmentation and dissolution of circumferential actin bundle, a structure crucial for maintenance of stable linear AJs. Simultaneously, we observed formation of dynamic lamellipodia containing branched actin network at the cell-cell boundaries and appearance of retrograde actin-myosin flow. We detected increased phosphorylation of the actin-binding protein EPLIN within minutes of the addition of EGF. It had been shown earlier that phosphorylation of EPLIN had resulted in its degradation through ubiquitin-proteasome-dependent mechanism [151]. It is known that EPLIN stabilizes circumferential actin bundle [13]. Thus, EGF-induced phosphorylation and degradation of EPLIN may lead to disruption of the circumferential actin bundle at the earliest stages of EMT. We also found that the early EMT-induced disruption of circumferential actin bundle was followed by transformation of the stable linear E-cadherin-based AJs into dynamic punctate AJs which were associated with straight actin bundles and co-localized with a tension-sensitive protein zyxin. The presence of zyxin indicated generation of centripetal forces at the cell-cell boundaries. Taken together, these observations reveal that early EMT promotes increased dynamics in the cell-cell contact areas, particularly the transformation of stable AJs and actin structures into dynamic ones. Contact paralysis—an essential property of the stable control AJs—disappeared, leading to weakening of cell-cell adhesion and disruption of cell-cell contacts. Cells released from the stable cell-cell contacts could then acquire front-rear polarity and eventually, a migratory phenotype. 

Front-rear polarity is an important characteristic of mesenchymal cells that defines directionality of migration. Front-rear polarity is a major actin cytoskeleton reorganisation; however, other cytoskeletal systems as well as polarity proteins contribute to its establishment. Generation of front-rear polarity is largely controlled by CDC42. Using stably expressed fluorescence resonance energy transfer (FRET) biosensors, it was found that in morphologically non-polarized cells, local activation of CDC42 and its spatial gradient drove the formation of initial protrusive fronts upon uniform chemotactic stimulation [152]. When the apical junctional complex was disrupted, CDC42 and the polarity protein complex PAR6-aPKC re-localized from the TJ region to the leading edge and induced re-localization of the centrosome and the Golgi apparatus to the front of the cell. Microtubule-organizing centers (MTOCs) associated with the centrosome and the Golgi apparatus promote microtubule (MT) growth towards the cell front, and subsequent cell migration and scattering [153,154]. MTs play a key role in the establishment and maintenance of front–rear polarity organizing MT-mediated intracellular transport, delivering of kinases and guanine nucleotide exchange factors (GEFs) for Rho GTPases to the leading edge [44,155]. MT-dependent delivery of mRNAs of the proteins that regulate the actin cytoskeleton and local translation also controls protrusion persistence in mesenchymal-like cells [156]. The EB1 protein localized at the plus ends of growing MTs recruits the CLIP-170-mDIA1 complex to accelerate actin filament elongation during lamellipodia formation [157].

In an in vitro model of EMT leading to epithelial cell scattering, it was found that protrusive activity at the free cell edges contributed to cell scattering via formation and attachment of integrin-mediated focal adhesions (FAs) to substrate and actomyosin contractility that transmitted to the rear cell-cell boundaries and caused disruption of the cell-cell contacts [158]. Integrins play an essential role in front-rear polarity and cell migration by engaging the Rho family of small GTPases (Rac, Rho and Cdc42) that coordinate cytoskeletal dynamics. Rho-dependent localization of myosin IIB at the cell rear is required for front-back polarity maintenance and tail retraction during mesenchymal migration [159,160].

## 7. Mesenchymal Migration

**Figure 5:**
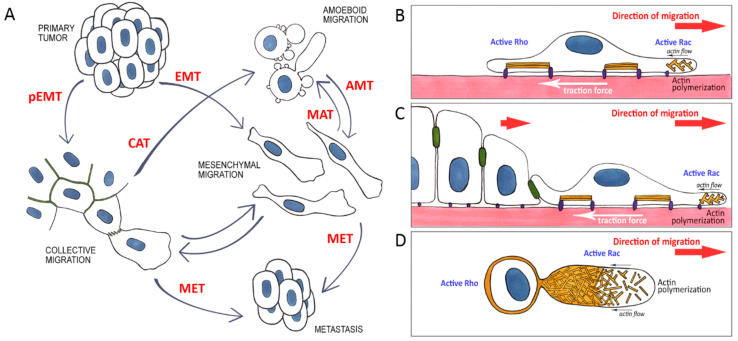
Plasticity of cancer cell migration. (**A**)—A primary epithelial tumor through partial epithelial- mesenchymal transition (pEMT) or epithelial-mesenchymal transtion (EMT) gives rise to motile cells capable of invasion via individual or collective mesenchymal migration. Specific microenvironment conditions govern reversible transitions between mesenchymal and amoeboid migration modes (collective-amoeboid transition (CAT), mesenchymal-amoeboid transition (MAT), amoeboid-mesenchymal transition (AMT)). In a distant metastasis, cells cease to migrate and revert to the original epithelial phenotype (mesenchymal-epithelial transition (MET)). (**B**–**D**)—Modes of cancer cell migration. Reorganized actin cytoskeleton and relative activity of Rho and Rac in the front and rear of the cells during individual (**B**), collective (**C**), or amoeboid (**D**) migration.

During metastasis, as was shown by intravital microscopy, cancer cells use different modes of migration such as mesenchymal migration of individual cells or groups of cells or amoeboid migration. This choice is dependent on substrate adhesiveness, composition of the extracellular matrix (ECM), activity of Rac and Rho GTPases regulating cytoskeletal dynamics, and MMP activity [161,162,163]. Among the MMPs, MT1-MMP plays a central role in pericellular matrix degradation [164]. Actin cytoskeleton dynamics is the basis for cell migration. In order to migrate, cells can use two properties of actin filaments: the ability to push by polymerization and the ability to contract by interacting with myosin (Figure 5).

Carcinoma cells use mesenchymal mode of migration moving along the BM, invading surrounding tissues or migrating in distant organs during metastatic colonization. Mesenchymal migration is characterized by repeating cycles that include extension of protrusions at the leading cell edge, their attachment to the substratum via FAs followed by detachment and retraction of the rear as a result of the cell contractility, which results in cell translocation. Migrating cells form flat protrusions (lamellipodia) and finger-like protrusions (filopodia). Extension of lamellipodia is driven by pushing forces generated by coordinated polymerization of a branched network of actin filaments oriented with their barbed ends towards the leading edge (Figure 1D, Figure 5B) [44,165,166]. This branched actin network is highly dynamic and is regulated by: (1) actin nucleators; (2) proteins promoting actin filament elongation; (3) capping proteins; (4) filament severing proteins [165,167]. The Arp2/3 complex consisting of seven subunits (Arp2, Arp3, ArpC1-C5) which nucleates a new actin filament off the side of a pre-existing filament plays the key role in formation of the branched actin network in lamellipodia [168]. The Arp2/3 complex requires activation by a nucleation-promoting factor WAVE that exists as a part of a pentameric protein complex [167,169]. The WAVE regulatory complex is recruited to lamellipodia by RAC1-GTP [170]. Ena/VASP proteins and formin family proteins binding with barbed ends of actin filaments promote their elongation [171,172,173]. Lamellipodin, which has been shown to interact both with the WAVE regulatory complex [174] and Ena/VASP proteins [175] in lamellipodia, can regulate lamellipodia dynamics and cell adhesion to ECM [176]. Actin filament debranching and severing is mediated by the actin depolymerizing factor (ADF)/cofilin family proteins [177].

Arp2/3 subunits overexpression signals poor prognosis for patients with lung, breast, and colorectal cancers. It is correlated with cancer progression, invasion and metastasis. 3D migration of cancer cells in vitro or in vivo has been shown to require Arp2/3 activity. Overexpression of the WAVE regulatory complex is observed in various carcinomas (breast, colon, liver, lung, ovary and prostate). Generally, overexpression of WAVE complex components is associated with reduced survival and lymph node invasion and metastasis. [178]. Lamellipodin has been shown to promote metastasis (specifically, tumor invasion and intravasation) in an orthotopic mouse breast cancer model, possibly through its interactions with Ena/VASP and WAVE. In breast cancer patients, moderate increase in Lamellipodin levels correlated with poorer prognosis [179]. Mena/VASP expression in breast, cervical, colorectal and pancreatis cancers correlated with high risk of metastases [180,181,182,183]. Mena invasion isoform promoted effective single-cell chain migration in mouse model of breast cancer [184].

Mesenchymal cells’ attachement to ECM via FAs is mediated by transmembrane heterodimeric receptors integrins. Small nascent adhesions constantly assemble at the edge of lamellipodia. Binding to ECM induces recruitment to FAs of paxillin, vinculin, and talin, which link integrins to actin filaments, leading to integrin activation and clustering [185]. Contractile forces generated by mutual sliding of actin and bipolar myosin II filaments induce growth and maturation of nascent adhesions into FAs. Cross-linking of actin filaments by α-actinin bundles the actin filaments associated with FAs into stress fibers and increases contractile forces (Figure 1D, Figure 5B) [186]. Actomyosin-mediated contraction of the cell body promotes disassembly of FAs at the cell rear, retraction of the rear and cell displacement [44,166]. FAs, being key components of cell migration machinery, play an important role in cancer cell dissemination. Integrins are involved in mechanotransduction through sensing the stiffness of the underlying surfaces and transmitting that information inside the cells, allowing them to adapt to their microenvironment. FAs contain multiple signaling molecules including FAK and Src family kinases, tyrosine phosphatases, and adaptor proteins [185]. It has also been found that integrin-mediated FAs accumulate regulators of RhoGTPase activity. It is known that the activity of Rho GTPases is regulated by guanine nucleotide exchange factors (GEFs), GTPase activating proteins (GAPs), and guanine nucleotide dissociation inhibitors (GDIs) [161,162,163,164,165,166,167,168,169,170,171,172,173,174,175,176,177,178,179,180,181,182,183,184,185,186,187]. Localization of GEFs and GAPs in different zones within cells results in local activation of Rho GTPases. Recently it has been found that integrin-based adhesions play a key role as multiprotein scaffolds spatially segregating GEFs and GAPs to promote RAC1 activation [188] which induces Arp2/3-dependent polymerization of the actin network and lamellipodia formation at the cell edge. 

Changes in expression patterns of integrins on cancer cells and in the production, secretion and remodeling of ECM by cancer cells and CAFs leading to increased tumor ECM stiffness, contribute to migratory behavior of cancer cells [189]. ECM stiffness affects localization and transcriptional activity of the mechanosencitive transcriptional co-factors YAP and TAZ [190,191]. ECM stiffness also promotes migration via FAK-dependent activation of RAC1 [192]. Integrin-specific signaling is essential for cancer progression. α6β4 intergin cooperates with oncogenic RTKs EGFR, ErbB2 while αvβ3 integrin cooperates with c-Met to amplify oncogenic signaling in tumors [193,194,195]. In cancer cells, integrin-specific signaling is also involved in pro-mitogenic and pro-survival signaling pathways such as the RAS/ERK, PI3K/AKT, and NF–kB (nuclear factor–kappa B) as has been thoroughly described in several reviews [196,197]. Integrin-dependent phosphorylation by Src and FAK of β-catenin and subsequent disruption of AJs contributes to weakening of cell-cell adhesion of pancreatic carcinoma cells growing on collagen type I [198]. Integrin-specific signaling is not restricted by focal adhesions. Upon endocytosis of integrins, FAK binds to and becomes activated on integrin-containing endosomes. Integrin endosomal signalling plays a key role in supporting anchorage-independent growth and anoikis resistance nessesary for survival in vasculature and formation of metastasis by cancer cells [199]. 

Using mesenchymal mode of migration, cells can move as single cells or as groups of cells, a process known as collective migration (Figure 5A–C). Cells of many types of carcinomas (e.g., ductal breast carcinoma, squamous cell carcinoma, colon carcinoma and others) can employ collective migration to invade adjacent tissues [200]. Collective migration is driven by front-rear polarization of leading cells that move using mesenchymal mode of migration and coordinate migration of the follower cells via signaling molecules and cadherin-based AJs [201]. At the invasion front, the lamellipodia of the leading cells generate traction forces on the substrate via integrins, while the cell–cell junctions transmit these forces from the front to the rear of the migrating group, which enables migration of follower cells [202]. The choice of a leader cell and establishment of its polarity is far from clear. The asymmetric distribution of cadherin-based adhesions may contribute to coordination of collective migration by restricting lamellipodial activity in the zones of cell-cell contacts and recruiting Rac GEFs at the leading cell edge [203]. Activation of leader cells depends on extracellular stimuli, such as ECM ligands or growth factors from microenvironment [201]. It was also demonstrated that cells behind the prospective leaders could locally increase traction forces to facilitate leader cell formation [204].

## 8. Amoeboid Migration

Amoeboid migration does not depend on integrin-based adhesions to the substratum and is driven by bleb-like protrusions sustained by high levels of Rho-mediated actomyosin contractility (Figure 5D). During amoeboid migration blebs grow as a result of intracellular pressure generated by actomyosin contraction pushing the membrane out in the regions where the cell membrane detaches from the cortex or where cortical actin exhibits local weaknesses [204,205,206,207]. Using amoeboid mode of migration, cancer cells can squeeze through pores in the ECM without requiring pericellular proteolysis [162,208]. Amoeboid migration is often faster than mesenchymal migration [207]. 

The molecular mechanisms controlling rearrangement of actin structures during active blebbing are not entirely clear. In expanding blebs of rounded cells membrane-associated proteins spectrin, adducin, ankyrin B1, myosins 1C and 1E have been found. Ezrin and moesin accumulate at the bleb membrane when expansion ceases and the actin cytoskeleton begins to assemble within the bleb by elongating cortical filaments and new filament polymerization driven by Arp2/3 and mDIA1 nucleation followed by α-actinin, tropomyosin, and myosin II accumulation [209,210]. In confined carcinoma cells that use amoeboid leader bleb-based migration, ERK-mediated EPS8 bundling activity modulates actin cortex and promotes cortex tension and intracellular pressure to drive bleb-based migration and leader bleb formation [211]. It remains to be elucidated how blebbing cells gain traction forces to promote forward cell translocation in the absence of adhesions at the leading edge. A study of migration of mammary carcinoma cells in Matrigel demonstrated that actin and myosin II could accumulate at the cell rear in a uropod-like structure and that β1 integrin was required for contraction of this uropod-like structure, which promoted cell migration [212]. It has been proposed that migrating cells can also employ non-specific interactions with surrounding substrate when rearward flowing actin cytoskeleton generates friction between cell and the substrate [213]. It is also suggested that in liquid, constant rearward plasma membrane flow generates tangential viscous forces that can propel the cell forward in the absense of any adhesion. RHOA-dependent increased endocytosis at the rear end of the cells and forward trafficking of membrane vesicles maintain the continuity of the membrane flow [214]. It has also been shown that melanoma cells migrating through compliant matrices form large blebs at the front which destroy the collagen matrix through mechanical interaction. The collagen is then internalized by the cells using macropinocytosis [215].

## 9. Mesenchymal-Ameboid Transition (MAT)

Phenotypic plasticity allows migrating tumor cells to pass through tissues with different molecular, structural and mechanical characteristics. Variations in substrate properties influence modes of cell migration (Figure 5A) [200,207,210]. Reduced cell–substrate adhesion favors the transition from mesenchymal to amoeboid phenotype. Studies of Walker 256 breast carcinosarcoma cell migration on micropatterned surfaces demonstrated that when cells moved from an adhesive to a non-adhesive substrate, lamellipodia could change to blebs and vice versa [216]. HT1080 fibrosarcoma cells cultured on a 2D adhesive substrate preferentially exhibited mesenchymal phenotype but on a substrate with reduced adhesiveness, a part of the cell population switched to forming blebs [217]. Cells switch to amoeboid migration under conditions of physical confinement and decreased adhesion [218]. Cells of breast and colon cancer cell lines migrating in 10-μm channels could switch between mesenchymal and amoeboid modes of migration. During migration in narrow 3-µm channels the same cells only exhibited blebbing at the leading edge. The transition from mesenchymal to amoeboid migration was accompanied by a loss of F-actin stress fibers. Cells passing through a narrow microchannel did not assemble FAs even on an adhesive substrate. A marked increase in cell velocity in narrow 3-μm channels compared to wider 10-μm channels was observed [219].

Plasticity of cell migration can be regulated via different signaling pathways. For example, MTLn3 mammary tumor cells overexpressing EGFR that possessed mesenchymal phenotype on adhesive substrates, exhibited amoeboid invasion both in vitro (3D collagen matrix) and in vivo (orthotopic injection into mice mammary fat pad) [220]. Met may also be involved in amoeboid cell motility. It has been demonstrated that MDA-MB-231 breast cancer cells expressing high levels of activated Met formed membrane blebs independently of HGF/SF and invaded three-dimensional matrix [221]. TGFβ signaling which has been shown to activate RHOA-dependent contractility [222] may contribute to MAT. In hepatocellular carcinoma cells, down-regulation of EGFR promoted TGFβ-induced transition to amoeboid invasion [223]. TGFβ also increased the number of MDA-MB-231 cells migrating in collagen matrices using amoeboid mode [224]. In some cases, hypoxia may be an important MAT-inducing factor. For example, 4T1 murine breast carcinoma cells that exhibited collective mesenchymal migration in collagen matrix in normoxia, in hypoxia switched to amoeboid migration. This switch may be connected to the downregulation of MMP observed in hypoxic conditions [225].

## 10. Conclusions

The studies summarized in this review demonstrate how pEMT may result in the cell phenotype diversity observed in cancers. Reversible changes in cytoskeleton and adhesive structure organization allow cancer cells to fine-tune their reaction to the microenvironment and change their phenotype and migratory properties to realize their invasive and metastatic potential. Further research is needed to shed more light on the molecular mechanisms that regulate these rearrangements. A deeper understanding of the EMT and plasticity of cell migration will help develop new therapeutic strategies to prevent cancer cell dissemination.

## Figures and Tables

**Table 1:** EMT transcription factors and their targets.

EMT-TF	Down-Regulated Expression	References	Up-Regulated Expression	References
SNAIL	E-cadherin Plakophilin-2 Claudin-4 Cytokeratins 17, 18, 19, 20 Gelsolin Occludin Integrins α3, α6, β4 Crumbs3	[46,47] [48] [48] [48] [48] [48,49,50,51] [52,53] [54]	Vimentin Claudin-11 MMP1 MMP2 MMP7 MT1-MMP Fibronectin Integrins α2, β1, β3	[55,56] [57] [56] [55,56,58] [56] [56] [56] [31,41,53]
SLUG	E-cadherin Occludin	[59,60] [50,61]	Vimentin Fibronectin	[60] [60]
TWIST1	E-cadherin α-catenin γ-catenin	[62,63,64] [62,63] [63]	N-cadherin Vimentin Smooth muscle actin Fibronectin Integrin α5	[64,65] [63,64,66] [62,63] [62,63] [67]
ZEB1	E-cadherin Occludin Crumbs3	[68,69] [70] [70]	*N*-cadherin Fibronectin Vimentin	[71] [71] [72]
ZEB2	E-cadherin α-catenin	[56,73] [73]	*N*-cadherin Vimentin MMP1 MMP2 MT1-MMP Fibronectin	[73] [56,74] [56] [56] [56] [56]

## Data Availability

No new data were created or analyzed in this study. Data sharing is not applicable to this article.
